# Hydrodynamic Simulation of an Orbital Shaking Test for the Degradation Assessment of Blood-Contact Biomedical Coatings

**DOI:** 10.3390/mi8040132

**Published:** 2017-04-19

**Authors:** Wen-Jin Cherng, Zuo-Syuan Dong, Chau-Chang Chou, Chi-Hsiao Yeh, Yu-Heng Pan

**Affiliations:** 1 College of Medicine, Chang Gung University, Taoyuan City 33302, Taiwan; cwenjin@cgmh.org.tw (W.-J.C.); yehccl@cgmh.org.tw (C.-H.Y.); 2 Division of Cardiology, Department of Internal Medicine, Chang Gung Memorial Hospital, Keelung 20401, Taiwan; 3 Department of Mechanical and Mechatronic Engineering, National Taiwan Ocean University, Keelung 20224, Taiwan; g00172032@gmail.com (Z.-S.D.), seohyun81714@gmail.com (Y.-H.P.); 4 Center for Marine Mechatronic Systems (CMMS), National Taiwan Ocean University, Keelung 20224, Taiwan; 5 Division of Thoracic and Cardiovascular Surgery, Chang Gung Memorial Hospital, Keelung 20401, Taiwan

**Keywords:** mechanical heart valve, computational fluid dynamics, degradation, biomedical coatings

## Abstract

Biomedical coatings are used to promote the wear resistance and the biocompatibility of a mechanical heart valve. An orbital shaking test was proposed to assess the durability of the coatings by the amount material eroded by the surrounding fluid. However, there is still a lack of understanding with regards to the shaker’s rotating conditions and the corresponding physiological condition. This study implemented numerical simulations by establishing a fluid dynamic model to evaluate the intensity of the shear stress under various rotating speeds and diameters of the shaker. The results are valuable to conduct in vitro tests for estimating the performance of biomedical coatings under real hemodynamic conditions and can be applied to other fluid-contact implants.

## 1. Introduction

Mechanical heart valves (MHVs) are widely used in the replacement of human heart valves and ventricular assist devices. In order to reduce the failure after valve transplantation, there is a biocompatible coating to modify the prostheses’ surface [1]. During the serving time of a MHV in vivo, its coating is constantly subject to the shear stress caused by the blood flow. In general, when the valves are moving from opening to closure, the average flow rate is greater than 0.8 m/s due to the Venturi effect [2,3,4]. The biocompatible coatings may lose their function and even be removed from the surface, of which the intensity of the shear stress and the duration are two major factors that cause the long-term degradation of the coatings.

To examine the adhesion strength between biocompatible coatings and a MHV, the samples with collagen–heparin composite coating were immersed in containing tubes filled with physiological saline, incubated at 37 °C, and then shaken on an orbital shaker for different durations [5]. The remaining material on the substrate was measured and compared. It is a simple and easy way to evaluate the long-term performance of any biomedical coating, organic or inorganic, on the blood contact device. However, there is still a lack of understanding with regards to the shaker’s rotating conditions, including the rotating speed and diameter, and the corresponding physiological condition.

In this work, an orbital shaker is chosen for simulating the fluid condition in heart and blood vessels; the remaining amount of coated heparin after the experiment is regarded as an indicator of the binding at different rotating speeds and diameters. A preliminary analysis of the biocompatible coatings degradation in dynamic condition is presented, and a previous experimental work was also investigated [6].

## 2. Materials and Methods

### 2.1. Problem Description

**Figure 1:**
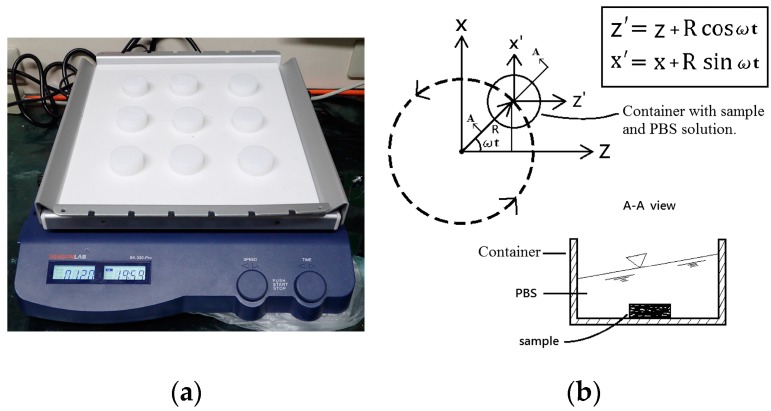
(**a**) The orbital shaker in this study. (**b**) The rotating path of the container, with a coated sample and phosphate-buffered saline (PBS) solution, and the relation equation of the fixed global coordinate (*X*, *Y*, *Z*) and the orbitally-moving coordinate (*X*’, *Y*, *Z*’). ω is the rotating speed in rad/s, *R* is the radius of the rotating path in mm, and t is the time in seconds. The A-A view is the cross section of the container to illustrate the detailed status of the fluid.

Figure 1a shows a commercially available orbital shaker. The container on the orbital shaker being translated along the path is illustrated in Figure 1b, where (*X*, *Y*, *Z*), (*X*’, *Y*, *Z*’), *R*, and ω represent the fixed global coordinate, the orbitally-moving coordinate on the container, the radius of the rotating path, and the rotating speed, respectively. As the sample and the fluid inside the container were both moving with the path at the same rotating speed, a simulation model was established by exploiting the moving boundary technique of the computational domain.

### 2.2. Simulation Setup

In this model, the computational area was composed of two domains: the surrounding air domain and the container domain. The dimensions and material properties of the domains are summarized in Table 1 and Table 2.

The simulation was carried out by ANSYS Fluent 14.5. The volume of fluid (VOF) model was adopted while the phase change and wall adhesion were not considered. The fluid had a multiphase property and a standard k-epsilon turbulence model was applied. The properties *P* of the fluid, including viscosity and density, were given as follows

(1) $$P=\alpha_{l}P_{l}+\left(1-\alpha_{l}\right)P_{g}$$

where the subscripts l and g are liquid and gas, i.e., the air here, and α, the volume fraction of the liquid phase.

The pressure drop across the fluid interface was expressed by the radius and the surface tension coefficient as follows

(2) $$p^{\prime }-p=\sigma \cdot \left(\frac{1}{r}+\frac{1}{r^{\prime }}\right)$$

where σ is the surface tension coefficient and the value was 0.072 N/m in this work; *p* and *r* are the pressure and the radius measured by the surface curvature in the normal direction to the interface; and *p*′ and *r*′ are the pressure and radius in the other phase.

The solution was modelled as a mixture of water and air, which was incompressible, viscous, and isothermal. The container’s walls were assumed to be rigid and under no-slip conditions. All results were regarded as convergent if the relative errors of residuals were less than 10^−4^. By means of this model, the shear flow was accessed in the flow.

Rotating diameter, rotating speed of the shaker, and the container’s inner diameter were three independent variables in the simulation. The rotating speed of the shaker varied from 60 rpm to 300 rpm, the rotating diameter of the shaker, 1 cm to 5 cm, and the container’s inner diameter, 2 cm to 5 cm, respectively. The meshing was composed primarily of tetrahedral mesh elements including hexahedral, pyramidal and wedge of various sizes. The element meshes were built in fine grids near the bottom of the containers and the liquid/gas interface. The total elements’ numbers were changed according to the size of the container, which were in the range of 21,020–65,970. Reynolds number (*Re*) is a measurement of the ratio of inertial force to viscous force in fluid hydrodynamics. Büchs et al. [7] developed the following equation to calculate the Reynolds number in shake flasks.

(3) $$Re=\;\frac{\rho \cdot n\cdot d^{2}}{\eta }$$

where ρ is the fluid density in [kg/m^3^], *n* is the shaking frequency in [s^−1^], *d* is the maximum inner diameter of the flasks, i.e., the container in this work, in [m], and η is the dynamic viscosity in [Pa·s]. When the container’s inner diameter was kept at 3 cm and the rotating speed of the shaker varied from 60 rpm to 300 rpm, i.e., 1 s^−1^ to 5 s^−1^, the values of Reynolds number were in the range of 900–4500. The k-epsilon turbulent equations are thus suitable for implementation in this study.

## 3. Numerical Results

**Figure 2:**
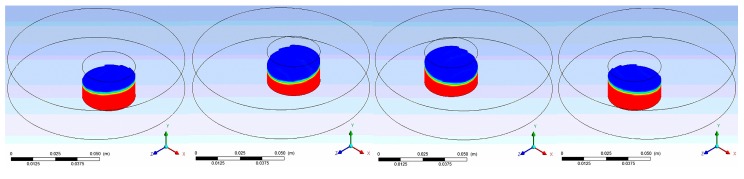
The shapes and surfaces (in blue) of the liquid (in red) in the container during a period, where the container is rotated at 120 rpm, i.e., the period is 2 s, along an orbital path with 1 cm diameter. For each position, from left to right, the simulating time is 0.625 s and the time increment is 0.5 s. The coordinate is *XYZ* system. (Container’s diameter = 3 cm)

**Figure 3:**
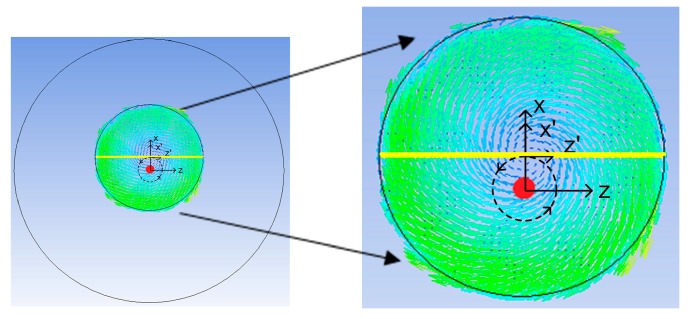
The average and maximum shear stresses in this work were all calculated along the yellow diameter which is perpendicular to the line connecting the origins of the global *XYZ* coordinate and the moving *X*’*YZ*’ coordinate.

In this section, we present the results of our numerical simulations for five rotating speeds, three rotating diameters of the shaker, and four inner diameters of the container. Figure 2 demonstrates a typical trajectory and liquid distribution at four positions where the shaker’s rotating speed, rotating diameter, and the container’s diameter are 120 rpm, 1 cm, and 3 cm. All the average and maximum shear stresses in this work were calculated along the yellow diameter of the container at any instant, as shown in Figure 3, which is perpendicular to the line connecting the origins of the global coordinate (*X*, *Y*, *Z*) and the moving coordinate (*X*’, *Y*, *Z*’). The numerical simulations were carefully studied in advance and the hydrodynamic values were evaluated only when the flow was under steady state.

### Dynamic Performance in the Rotational Speed and Diameter Variation

To evaluate the variable hydrodynamic condition in terms of the erosion from the flow acting on the surface of a MHV, the instantaneous viscous shear stresses can be obtained by the CFD model established in this work.

**Figure 4:**
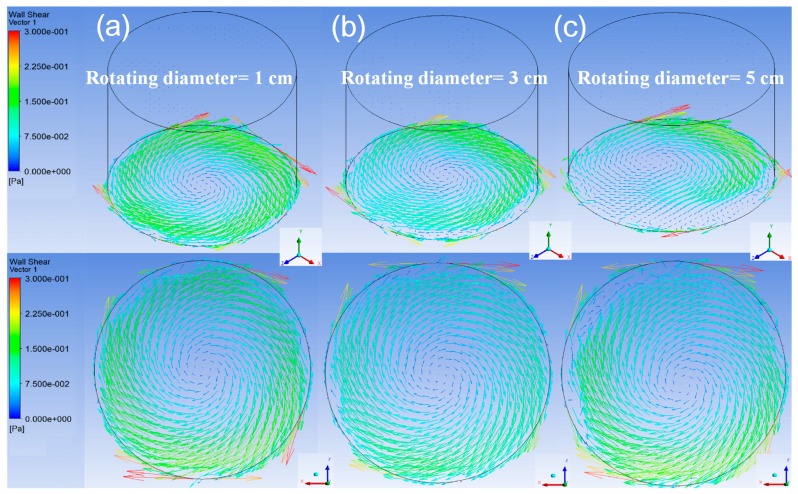
The isometric view (upper) and top view (lower) of the shear stress distributed on the bottom surface of the container. The shaker’s rotational speed is 120 rpm and the rotational diameter is (**a**) 1 cm, (**b**) 3 cm, and (**c**) 5 cm, respectively. The coordinate is *X*’*YZ*’ system. (Container’s diameter = 3 cm)

**Figure 5:**
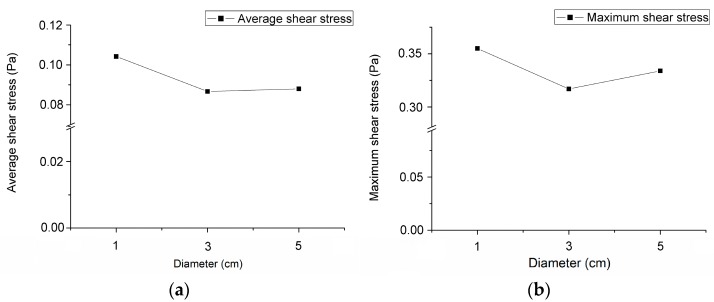
The variation of (**a**) the average shear stress, and (**b**) the maximum shear stress along the specific diameter as defined in Figure 3 on the bottom surface of the container with three rotating diameters. (Rotating speed = 120 rpm and container’s diameter = 3 cm)

After a very short transient procedure, the flow inside the container was proven, from period to period, to be steady and well-posed at any specific position of the orbital path as indicated in Figure 2. The distributions of the shear stress vector for three rotating diameters on the bottom surface at the simulating time of 2.5 s are visualized in Figure 4 by isometric view and top view. The rotating speed of these cases was kept at 120 rpm. The average and maximum shear stresses along an arbitrary diameter on the bottom surface of the container are shown in Figure 5. The distributions of the shear stress are similar, which suggests that the rotating diameter has little effect on the shear stress of the bottom surface.

**Figure 6:**
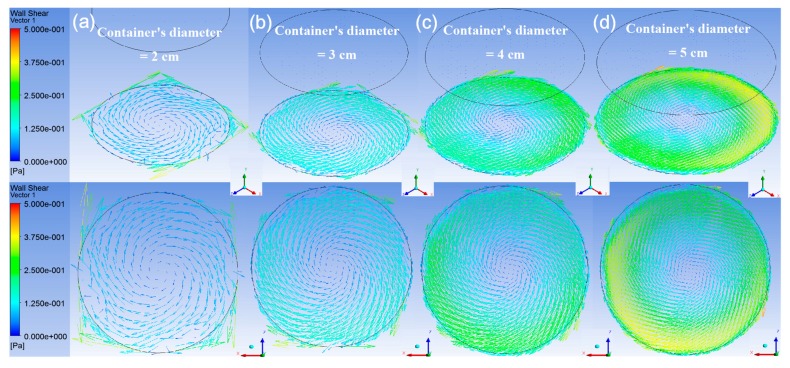
The isometric view and top view of the shear stress distribution on the bottom surface of the container. The shaker’s rotational diameter and the rotational speed are 1 cm and 120 rpm. The inner diameter of the container is adjusted to be (**a**) 2 cm, (**b**) 3 cm, (**c**) 4 cm, and (**d**) 5 cm respectively from left to right. The length of the arrow scale on each image changes with the inner container’s diameter. The coordinate is *X*’*YZ*’ system.

**Figure 7:**
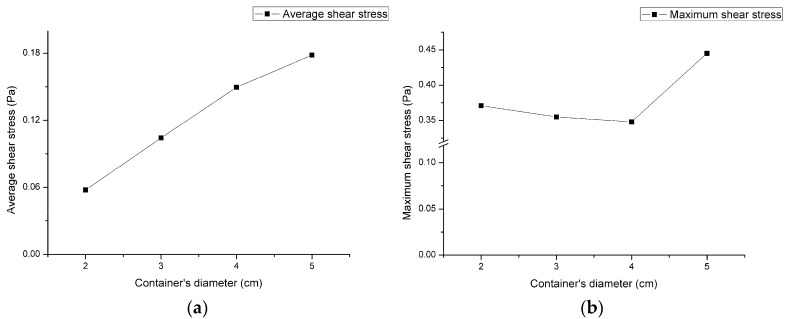
The variation of (**a**) the average shear stress, and (**b**) the maximum shear stress along the specific diameter as defined in Figure 3 on the bottom surface of the container with four containers’ diameters. (Rotating diameter = 1 cm and rotating speed = 120 rpm)

To investigate the influence of the container’s dimensions on the shear stress over the sample’s surface, the distributions of the shear stress vectors for four containers’ diameters on the bottom surface at the simulating time of 2.5 s are illustrated by isometric view and top view in Figure 6. The rotating diameter and rotating speed of these cases were kept at 1 cm and 120 rpm. The average stresses and maximum stresses are shown in Figure 7. In Figure 7a, the average stress increases linearly with the container’s diameter; however, the maximum stress in Figure 7b retains its value until the container’s diameter becomes 5 cm.

**Figure 8:**
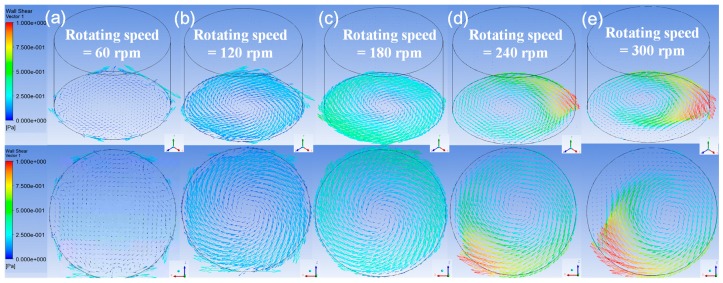
The isometric view and top view of the shear stress distribution on the bottom surface of the container. The shaker’s rotational diameter is fixed at 1 cm and the rotational speed is adjusted to (**a**) 60 rpm, (**b**) 120 rpm, (**c**) 180 rpm, (**d**) 240 rpm, and (**e**) 300 rpm respectively from left to right. The coordinate is *X*’*YZ*’ system. (Container’s diameter = 3 cm)

**Figure 9:**
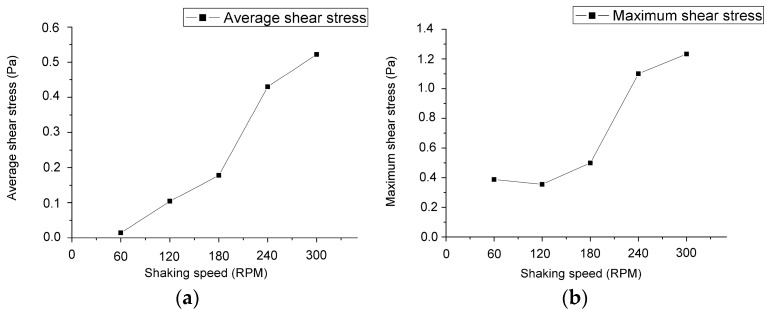
The variation of (**a**) the average shear stress and (**b**) the maximum shear stress along the specific diameter defined in Figure 3 on the bottom surface of the container with five rotating speeds. (Rotating diameter = 1 cm and container’s diameter = 3 cm)

In Figure 8, the distributions of the shear stress vectors for three rotating speeds on the bottom surface at the simulating time of 2.5 s are also illustrated by isometric view and top view. The rotating diameter and container’s diameter of these cases were kept at 1 cm and 3 cm. The average stresses and maximum stresses are shown in Figure 9. It is worth noting that the trend of the stresses is more significant than those in Figure 5 with the rotating diameters and those in Figure 7 with the container’s diameters, especially at the rotating speeds between 180 and 240 rpm. Therefore, the rotating speed of the orbital shaker is suggested to be the major control parameter to set up the shear stress level of the long-term erosion test in this study.

## 4. Discussion

### 4.1. An Experimental Application

**Figure 10:**
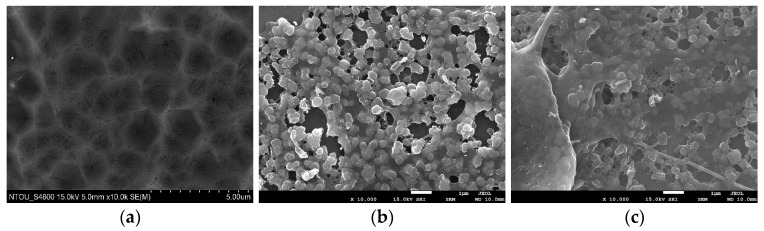
Surface images (×10 k) taken by scanning electron microscopy from (**a**) bare titanium alloy; (**b**) titanium alloy sequentially coated with one dopamine interlayer, four heparin/collagen multilayers, and one heparin outmost layer, Ti/Dop/(Hep_Col)_4_/Hep; and (**c**) titanium alloy, with one dopamine interlayer, nine heparin/collagen multilayers, and one heparin outmost layer, Ti/Dop/(Hep_Col)_9_/Hep.

**Figure 11:**
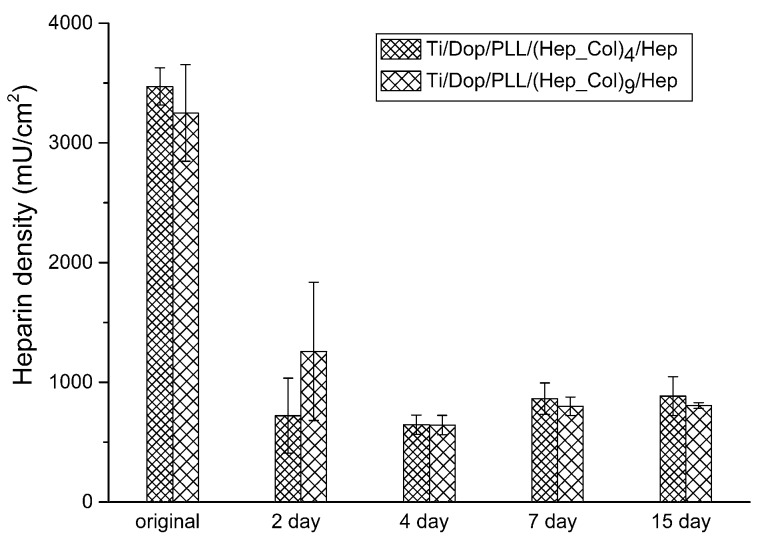
Heparin density of two samples, Ti/Dop/(Hep_Col)_4_/Hep and Ti/Dop/(Hep_Col)_9_/Hep, from the beginning to the end of various periods of the tests conducted by the orbital shaker (*N* = 3).

Functional coatings are very popular and effective in biomedical applications. The authors recently developed a new treatment [6] to improve the adhesion and blood-compatibility of the titanium substrate (Ti) coated with heparin/collagen multilayers (Hep_Col). The adhesion strength of the layer-by-layer coatings was evaluated by the scratch tests of a sliding stylus, which cannot reveal the degradation of the coated materials after a long-term erosion by the contact fluid. The orbital shaking test is believed to be a more practical and convenient way to survey the durability of biomedical coatings. The anticoagulant, heparin (Hep), of the multilayers in this work was measured by the toluidine blue O (TBO) assay and the details were reported in [8]. The surface topographic images of bare titanium alloy and the sample sequentially coated with one dopamine interlayer (Dop), four heparin/collagen multilayers, and one heparin outmost layer, Ti/Dop/(Hep_Col)_4_/Hep; and that with nine heparin/collagen multilayers and one heparin outmost layer, Ti/Dop/(Hep_Col)_9_/Hep, were shown in Figure 10. Figure 11 demonstrates the quantitative analysis of the coatings by the remaining heparin from the beginning to the end of a fifteen-day period. The container’s diameter was 3 cm. The rotating speed of the orbital shaker was kept at 120 rpm and the rotating diameter, 1 cm. It can be observed that the heparin/collagen coatings were significantly removed by the fluid for the first two days. The samples with nine multilayers, even with almost the same heparin at the beginning, could maintain higher heparin content. However, when the tests exceeded four days, the degradation of both coatings reached a low and steady condition until the fifteen-day stage. According to the numerical results in this study, the average shear stress and maximum shear stress actuated on the coatings were 0.1 and 0.4 Pa, which can be assessed and compared with the real physiological conditions.

### 4.2. Limitations and Advantages of the Test

For a MHV, the real physiological flow is a pulsitile one and is sometimes acompanied by jets during opening and closing stages. A bileaflet valve, the most popular type, has forward jets during the opening stage, and periphery, b-datum, and hindge reverse jets during the closing stage [9]. The instantaneous intensity of the maximum shear stress in various flowfields was widely suggested by different approaches. The values were reported to be 90–150 Pa along the centreline plane at peak systole downstream of the valve [9,10], while other groups portrayed smaller values, 6–6.4 Pa [3,11], instead. Regardless of the variation in the values reported, they are still valuable to evaluate the different flow fields in terms of the forces acting on blood cells. However, the shear stress actuating on the surfaces of the MHVs during the whole pulsatile process is still not clearly discussed. The orbital shaking test implemented in this work focused on the constant erosion effect on the adhesion of the coating of a blood contact prosthesis. The maximum shear stress in this study was limited by the commercially available shaker but the method is still promising to provide a much higher intensity once another test rig is built. This work demonstrated a new approach to compare and refine the possible coating candidates in a simple and fast manner. It is suitable for the preliminary development of the coating technique. Nevertheless, for a more intensive study, a pulsatile circulation system should be conducted.

## 5. Conclusions

As the degradation of the biomedical coatings is caused by the shear stress and the actuating duration, this work provides a numerical evaluation of the shear stress in the container of an orbital shaker to investigate the mechanism of the degradation. The rotational speed of the shaker is verified to be the major operating parameter that can change the shear stress of the flow in the container. By applying the results to the experimental investigation, the correlation of the biomedical coating’s degradation in the fluid-contact conditions and its shear stress levels were demonstrated. This work demonstrates a convenient way for the preliminary development of the coating materials and their building technique.

## Figures and Tables

**Table 1:** Properties of the surrounding air domain.

**Diameter [mm]**	100
**Height [mm]**	20
**Density [kg/m^3^]**	1.225
**Dynamic Viscosity [kg/(s·m)]**	0.00001789

**Table 2:** Properties of the container domain.

**Diameter [mm]**	30
**Height [mm]**	20
**Liquid level [mm]**	10
**Air Density [kg/m^3^]**	1.225
**Solution Density [kg/m^3^]**	998.2
**Solution Dynamic Viscosity [kg/(s·m)]**	0.001003

## References

[1] Chen J., Li Q., Chen J., Chen C., Huang N. (2009). Improving blood-compatibility of titanium by coating collagen–heparin multilayers. Appl. Surf. Sci..

[2] Huang C.-K., Chen J.-H. (2013). A comparison study of cavitating flow in a ventricular assist device using laminar and turbulent model. JFCMV.

[3] Borazjani I., Sotiropoulosa F. (2010). The effect of implantation orientation of a bi-leaflet mechanical heart valve on kinematics and hemodynamics in an anatomic aorta. J. Biomech. Eng. Trans. ASME.

[4] Jones S.A. (1995). A relationship between Reynolds stresses and viscous dissipation: Implications to red cell damage. Ann. Biomed. Eng..

[5] Chen J., Huang N., Li Q., Chu H., Li J., Maitz M.F. (2016). The effect of electrostatic heparin/collagen layer-by-layer coating degradation on the biocompatibility. Appl. Surf. Sci..

[6] Chou C.-C., Hsin S.-W., Lin H.-C., Yeh C.-H., Wu R., Cherng W.-J. (2016). Oxidized dopamine as the interlayer between heparin/collagen polyelectrolyte multilayers and titanium substrate: An investigation on the coating’s adhesion and hemocompatibility. Surf. Coat. Technol..

[7] Büchs J., Maier U., Milbradt C., Zoels B. (2000). Power consumption in shaking flasks on rotary shaking machines: I. Power consumption measurement in unbaffled flasks at low liquid viscosity. Biotechnol. Bioeng..

[8] Liu T., Liu Y., Chen Y., Liu S., Maitz M.F., Wang X., Zhang K., Wang J., Wang Y., Chen J. (2014). Immobilization of heparin/poly-(l)-lysine nanoparticles on dopamine-coated surface to create a heparin density gradient for selective direction of platelet and vascular cells behavior. Acta Biomater..

[9] Dasi L.P., Simon H.A., Sucosky P., Yoganathan A.P. (2009). Fluid mechanics of artificial heart valves. Clin. Exp. Pharmacol. Physiol..

[10] Xenos M., Girdhar G., Alemu Y., Jesty J., Slepian M., Einav S., Bluestein D. (2010). Device Thrombogenicity Emulator (DTE): Design optimization methodology for cardiovascular devices: A study in two bileaflet MHV designs. J. Biomech..

[11] Hedayat M., Asgharzadeh H., Borazjani I. (2017). Platelet activation of mechanical versus bioprosthetic heart valves during systole. J. Biomech..

